# A Comprehensive Review of Monoclonal Antibodies in Modern Medicine: Tracing the Evolution of a Revolutionary Therapeutic Approach

**DOI:** 10.7759/cureus.61983

**Published:** 2024-06-09

**Authors:** Manjeet Kothari, Anil Wanjari, Sourya Acharya, Vineet Karwa, Roma Chavhan, Sunil Kumar, Ajinkya Kadu, Rajvardhan Patil

**Affiliations:** 1 Medicine, Jawaharlal Nehru Medical College, Datta Meghe Institution of Higher Education and Research, Wardha, IND

**Keywords:** prospects, clinical successes, modern medicine, evolution, therapeutic applications, monoclonal antibodies

## Abstract

Monoclonal antibodies (mAbs) have emerged as potent therapeutic agents, revolutionizing the landscape of modern medicine. This comprehensive review traces the evolution of mAbs from their inception to their current prominence, highlighting key milestones in their development and exploring their diverse therapeutic applications. Beginning with an overview of their molecular structure and mechanisms of action, we delve into the production and engineering of mAbs, including hybridoma technology and recombinant DNA techniques. Therapeutic applications across various medical disciplines, including cancer treatment, autoimmune diseases, and infectious diseases, are examined in detail, showcasing the significant clinical successes of mAbs. Furthermore, this review discusses the challenges and opportunities in manufacturing scalability, cost-effectiveness, and access to therapies. Looking ahead, the implications of mAbs in future research and clinical practice are explored, emphasizing the potential for next-generation mAbs, personalized medicine, and integration with emerging modalities such as immunotherapy and gene therapy. In conclusion, the evolution of monoclonal antibodies underscores their transformative impact on healthcare and their continued promise to advance the frontiers of medicine.

## Introduction and background

Monoclonal antibodies (mAbs) are laboratory-produced molecules engineered to mimic the immune system's ability to fight off harmful pathogens and diseased cells [1]. These antibodies are designed to target specific antigens with high precision, offering a potent and targeted therapeutic approach for various medical conditions [2]. Monoclonal antibodies have revolutionized medicine by offering highly effective treatments for various diseases, including cancer, autoimmune disorders, infectious diseases, and more. Their specificity and versatility make them invaluable diagnostic and treatment tools, providing clinicians with powerful options to combat complex medical challenges [3].

This comprehensive review explores monoclonal antibodies' evolution and the current landscape in modern medicine. By tracing their historical development, elucidating their structure and function, and examining their therapeutic applications, this review provides a thorough understanding of the significance of mAbs in clinical practice. Furthermore, the review will discuss the clinical successes, challenges, and prospects of monoclonal antibodies, offering insights into their role in shaping the future of medicine.

## Review

Historical background

Discovery of Antibodies

The historical narrative surrounding the discovery of antibodies is rich and spans significant milestones in immunology and medicine. Antibodies, characterized as Y-shaped proteins synthesized by B cells, are essential in the body's immune response against pathogens such as bacteria and viruses [4,5]. In 1890, Emil von Behring and Shiba Saburo Kitasato demonstrated the efficacy of serum therapy in treating diphtheria, marking a pivotal moment in antibody research [4,5]. German researcher Paul Ehrlich coined the term "antibody" in 1897, defining antibodies as branched molecules that bind to toxins, laying the groundwork for understanding their role in immunity [6,7]. In 1959, Rodney Porter and Gerald Edelman independently unveiled the molecular structure of antibodies, a groundbreaking feat that earned them the Nobel Prize in Physiology or Medicine [7]. The invention of the ELISA test in 1971 by Swedish researchers Eva Engvall and Peter Perlman revolutionized antibody identification and measurement, facilitating the detection of hormones and viruses within the body [7]. Japanese researcher Susumu Tonegawa, who was awarded the Nobel Prize in Physiology or Medicine in 1987, unraveled the mystery of how a limited set of genes can produce a vast array of antibodies via the random rearrangement of genes [7]. These milestones underscore the progression in our comprehension of antibodies, transitioning from their early therapeutic application in serum therapy to elucidating their molecular structure and genetic underpinnings. This trajectory has paved the way for developing monoclonal antibodies and their diverse applications in modern medicine [6,7].

Development of Hybridoma Technology

Köhler and Milstein introduced hybridoma technology in 1975, a pioneering advancement in biotechnology that has since transformed the landscape of monoclonal antibody production [8]. This innovative technique involves the fusion of antibody-producing B cells with immortal myeloma cells, resulting in hybrid cells termed hybridomas capable of continuous, high-yield production of specific antibodies [8]. Hybridoma technology has revolutionized the field by facilitating the creation of large quantities of identical antibodies, known as monoclonal antibodies. The process commences with immunizing a mammal, typically a mouse, with a specific antigen to trigger an immune response. Subsequently, antibody-producing B cells are extracted from the immunized animal and fused with myeloma cells, generating hybridomas. These hybridomas can be cultured to produce monoclonal antibodies that exhibit chemical identity and high specificity toward the targeted antigen [9]. The advent of hybridoma technology has had a profound impact on immunology and biomedical research, offering a systematic approach to generating monoclonal antibodies with unparalleled specificity and uniformity. This technique finds extensive applications across diverse fields, including therapeutics, disease detection, targeted therapies, and experimental investigations. Hybridoma-derived monoclonal antibodies serve as precise tools for detecting and analyzing specific biomolecules, thereby enhancing our understanding of biological processes [10].

Milestones in Monoclonal Antibody Research

Köhler and Milstein introduced hybridoma technology in 1975, a pioneering advancement in biotechnology that has since transformed the landscape of monoclonal antibody production [11]. This innovative technique involves the fusion of antibody-producing B cells with immortal myeloma cells, resulting in hybrid cells termed hybridomas capable of continuous, high-yield production of specific antibodies [12-16]. Hybridoma technology has revolutionized the field by facilitating the creation of large quantities of identical antibodies, known as monoclonal antibodies. The process commences with immunizing a mammal, typically a mouse, with a specific antigen to trigger an immune response. Subsequently, antibody-producing B cells are extracted from the immunized animal and fused with myeloma cells, generating hybridomas. These hybridomas can be cultured to produce monoclonal antibodies that exhibit chemical identity and high specificity toward the targeted antigen [17]. The advent of hybridoma technology has had a profound impact on immunology and biomedical research, offering a systematic approach to generating monoclonal antibodies with unparalleled specificity and uniformity. This technique finds extensive applications across diverse fields, including therapeutics, disease detection, targeted therapies, and experimental investigations. Hybridoma-derived monoclonal antibodies serve as precise tools for detecting and analyzing specific biomolecules, thereby enhancing our understanding of biological processes [18-20].

Structure and function of monoclonal antibodies

Molecular Structure

Monoclonal antibodies have a "Y-shaped" structure composed of four polypeptide chains: two identical heavy chains and two identical light chains. The total molecular weight of a monoclonal antibody is around 150 kDa. The two arms of the "Y" are called the Fab (antigen-binding fragment) regions, which contain the variable domains responsible for antigen binding. The stem of the "Y" is called the Fc (fragment crystallizable) region, which determines the class/isotype of the antibody and mediates effector functions. The Fab region contains complementarity-determining regions (CDRs) that allow the antibody to bind to a specific antigen epitope with high affinity. The heavy chain forms the lower part of the "Y" and is the constant region, while the light chain forms the upper arms and is the variable region responsible for antigen binding [15-18].

Mechanisms of Action

Signaling-mediated cell death: Cross-linking surface antigens can induce cell death. Monoclonal antibodies (mAbs) can induce cell death, initiating apoptotic signaling pathways. Blocking activation signals: Through their action, mAbs can intercept growth-promoting signals, effectively halting the continuous proliferation of tumor cells. Antibody-dependent cellular cytotoxicity (ADCC): By binding to Fc receptors on immune cells such as Natural Killer (NK) cells, mAbs can instigate ADCC, leading to the targeted killing of designated cells. Complement-mediated cytotoxicity (CMC): The Fc region of mAbs can activate the complement system, thereby instigating the formation of the membrane attack complex and ensuing lysis of target cells. Modulating the cytokine environment: Through their influence, mAbs can modulate the cytokine milieu, amplifying anti-tumor immune responses. Agonism of immune receptors: Certain mAbs can act as agonists, activating receptors like ICOS on T cells to promote anti-tumor immunity. Antagonism of ligands/receptors: mAbs have the potential to obstruct interactions between tumor cells and growth factors or receptors, thereby impeding tumor growth and promoting cellular survival [19,20].

Variability in Specificity and Affinity

The specificity and affinity of monoclonal antibodies (mAbs) can vary significantly due to multiple factors. Research indicates that mAbs may exhibit a spectrum of binding specificities, ranging from highly specific to highly cross-reactive, with some mAbs demonstrating reactivity with multiple normal tissues [21]. For instance, a study involving 52 thoroughly characterized mAbs targeting carcinoembryonic antigen (CEA) revealed various binding patterns. Most mAbs exhibited cross-reactivity with various normal tissues, with some being highly cross-reactive (positively reacting with 8 out of 9 discriminating tissues) and others showing relatively specific reactions (positively reacting with only 1 out of 9 discriminating tissues). A mere 10% of the mAbs were identified as specific, reacting solely with colon carcinoma, normal colon mucosa, and normal gastric foveola [21]. Furthermore, the study determined the binding constant for the mAb-CEA interaction, revealing no direct correlation between antibody specificity and affinity for CEA. This suggests that mAbs with high specificity may demonstrate varying levels of affinity, and conversely, mAbs with high affinity may not always exhibit high specificity [21]. Moreover, research underscores the importance of balancing affinity and non-specific binding when optimizing mAbs for therapeutic purposes. Investigations have shown that while most antibodies with high affinity tend to display relatively high non-specific binding, those with substantial reductions in non-specific binding often experience a decrease in affinity [22]. Thus, optimizing mAbs for therapeutic use necessitates careful consideration of these trade-offs between affinity and non-specific binding.

Production and engineering of monoclonal antibodies

Hybridoma Technology

Hybridoma technology is a well-established method to produce monoclonal antibodies (mAbs) tailored to specific antigens of interest [23]. This technique entails the fusion of a short-lived antibody-producing B cell with an immortal myeloma cell, culminating in the formation of hybridoma cell lines. Each distinct hybridoma cell line is characterized by the robust expression of a singular specific mAb, thereby facilitating the continuous production of identical antibodies. Originating in 1975, this groundbreaking technology was pioneered by Georges Köhler and César Milstein, who adeptly fused normal B cells from immunized mice with myeloma cells. The outcome was the creation of immortal hybrid cells capable of generating antibodies precisely targeted to particular antigens. The advent of hybridoma technology has left an indelible mark on biotechnology, furnishing researchers and clinicians with a potent tool for generating monoclonal antibodies. These antibodies find application across diverse domains, encompassing research, diagnostics, and therapeutic interventions [24].

Recombinant DNA Technology

Recombinant DNA technology encompasses the intricate process of amalgamating DNA fragments from disparate sources, including distinct species, to generate novel genetic sequences that do not occur naturally [25]. This methodology involves a series of meticulously orchestrated steps: isolation of the desired DNA, precise cleavage at predetermined recognition sites utilizing restriction enzymes, amplification of gene copies via polymerase chain reaction (PCR), ligation of DNA fragments into a vector, and subsequent insertion of the recombinant DNA into a host organism [26]. The advent of recombinant DNA technology facilitates the production of copious amounts of specific proteins, modifying existing genes, and incorporating foreign genes into organisms. Such capabilities find extensive application in diverse realms, spanning medicine, agriculture, and industrial biotechnology [27]. Notable applications of recombinant DNA technology encompass the production of therapeutic proteins (e.g., insulin, growth hormones), gene therapy, genetic manipulation of crops, and the creation of diagnostic tools such as ELISA [27]. The ability to manipulate DNA sequences has catalyzed a paradigm shift in our comprehension of genetics and has spearheaded significant advancements in molecular biology, genomics, and proteomics [27].

Transgenic Mice and Phage Display

Two pivotal technologies utilized in the production and engineering of monoclonal antibodies are transgenic mice and phage display. Transgenic mice are genetically modified to express complete human antibody repertoires, facilitating the rapid and efficient generation of human antibodies boasting enhanced affinity and specificity. These mice serve as invaluable platforms for in vivo affinity maturation, culminating in the synthesis of fully human antibodies characterized by reduced immunogenicity and heightened therapeutic efficacy [28,29]. In contrast, phage display technology entails the construction of diverse libraries housing antibody-variable regions displayed on phage surfaces. This innovation enables swift screening and selection of monoclonal antibodies specific to targeted antigens. Phage display serves as a potent alternative to traditional hybridoma technology, facilitating the identification of human-derived antibodies tailored to specific antigens and offering a valuable tool for antibody discovery and refinement [28,29]. Both transgenic mice and phage display platforms have made substantial contributions to the advancement of monoclonal antibodies in modern medicine. While transgenic mice excel at facilitating in vivo affinity maturation and the production of fully human antibodies, phage display technology provides a versatile and efficient avenue for identifying target-specific antibodies from diverse libraries of antibody variable regions [28,29].

Therapeutic applications of monoclonal antibodies

Cancer Treatment

Monoclonal antibodies (mAbs) employ various mechanisms to combat cancer cells, each tailored to elicit a targeted immune response. First, certain mAbs trigger the immune system, prompting it to recognize and attack cancerous cells. By binding to proteins present on the surface of cancer cells, these mAbs enhance the identification process for immune cells, facilitating their destruction of abnormal cells through antibody-dependent cell-mediated cytotoxicity (ADCC) [30,31]. Alternatively, some mAbs function by intercepting signals that promote cancer cell division. By blocking these signals, mAbs impede the proliferation and dissemination of cancer cells, offering a focused approach to treatment [30,31]. Another strategy involves conjugating mAbs with chemotherapy drugs or radioactive particles. Acting as targeting agents, these mAbs deliver the attached substances directly to cancer cells, enhancing the precision and efficacy of treatment by concentrating toxic agents precisely where they are needed most [30,31]. Moreover, bispecific T-cell engagers (BiTEs) represent a novel class of mAbs designed with dual functionality. One segment of the BiTE attaches to a protein present in cancer cells, while the other segment binds to T cells, a type of immune cell. This interaction orchestrates a potent immune response against cancer cells, augmenting the body's ability to combat the disease [30,31].

Autoimmune Diseases

The utilization of monoclonal antibodies (mAbs) in treating autoimmune diseases represents a significant stride forward in therapeutic options. These interventions primarily focus on specific molecules expressed during B-cell development, including CD20, CD19, CD22, CD38, and B-cell activating factor (BAFF) [32]. By binding to antigens, ligands, or receptors on B cells, mAbs disrupt downstream signaling pathways linked to cellular growth, proliferation, or apoptosis [33]. Various mechanisms drive the apoptosis of target cells induced by different mAbs, ranging from direct induction to indirect elimination via antibody-dependent cell-mediated cytotoxicity (ADCC) and complement-dependent cytotoxicity (CDC) [33]. However, the use of mAbs in autoimmune diseases can give rise to various complications, such as the development of autoantibodies, lupus-like syndromes, and conditions like cutaneous or systemic vasculitis, nephritis, and demyelinating syndromes, along with thyroid autoimmunity [34,35]. For example, mAbs targeting TNF for rheumatic diseases have been associated with the emergence of antinuclear antibodies, antibodies to double-stranded DNA, and lupus-like syndromes [35]. Similarly, mAbs like alemtuzumab, employed in treating multiple sclerosis, can induce antibody-mediated thyroid autoimmunity [35]. Despite these challenges, the therapeutic landscape for autoimmune diseases is rapidly evolving, with over 350 clinical trials underway to explore combined mAb therapy, identify new targets, examine interactions in the tumor-host microenvironment, and delineate biomarkers associated with response and resistance [34]. Nevertheless, the development of neutralizing anti-monoclonal antibodies can lead to therapeutic response loss and resistance to current mAb treatments, necessitating further investigation to optimize treatment duration and identify patient subgroups suitable for discontinuation of treatment [34]. Monoclonal antibodies have emerged as potent treatments for autoimmune diseases, offering targeted therapeutic alternatives with the potential for substantial clinical advantages. However, the intricate nature of autoimmune conditions and the possibility of adverse effects underscore the criticality of continuous research aimed at refining treatment strategies and enhancing patient outcomes [34].

Infectious Diseases

The utilization of monoclonal antibodies (mAbs) in the realm of infectious diseases stands as a significant domain of research and application within modern medicine. These meticulously crafted antibodies are engineered to target specific components of viruses or bacteria, effectively thwarting their ability to infiltrate cells or disrupting their capacity to inflict harm or provoke infection [36]. Their deployment has spanned various infectious diseases, encompassing ailments such as influenza, Ebola, Zika, and dengue, and, notably, serving as a critical asset in the battle against SARS-CoV-2 (COVID-19) and respiratory syncytial virus (RSV) [36]. In the context of infectious diseases, mAbs offer a promising avenue for both prophylaxis and treatment, demonstrating efficacy in curtailing viral load and mitigating the severity of illness [36-38]. The adaptability and precision targeting prowess of mAbs render them potent allies in the fight against infectious agents, with ongoing exploration to unearth their potential applications across a broad spectrum of pathogens [36-38].

Other Emerging Therapeutic Areas

**Figure 1:**
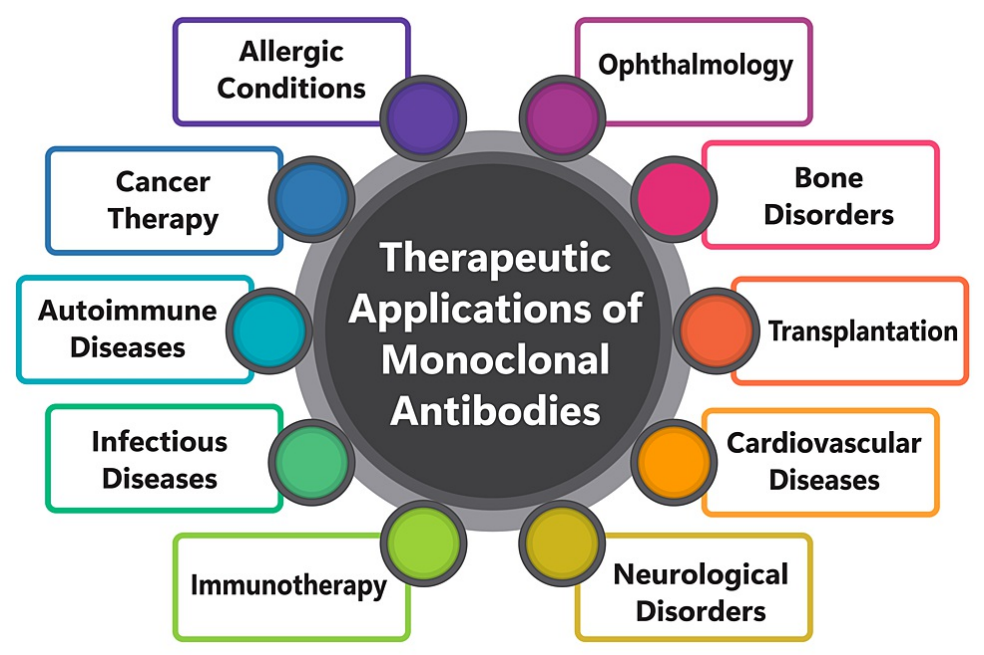
Shows therapeutic applications of monoclonal antibodies The image was created by the corresponding author.

Monoclonal antibodies and antibody-drug conjugates are emerging as promising therapeutics for breast cancer treatment, targeting specific receptors and pathways involved in tumor growth [39]. These innovative approaches hold immense potential for combating breast cancer by precisely targeting the underlying mechanisms driving tumor progression. Additionally, novel antibody derivatives, including antibody fragments, bispecific antibodies, and antibody-drug conjugates, are under development to diagnose and treat malignant tumors within the intricate tumor microenvironment [40]. These advancements represent a multifaceted approach to tackling the complexities of malignant tumors, offering tailored solutions for diagnosis and treatment. Moreover, advances in biotechnology are spearheading the development of precise tumor immunotherapy utilizing novel antibodies, such as TCR mimic antibodies designed to target proteins inside tumor cells [40]. These cutting-edge therapies hold promise for revolutionizing cancer treatment by leveraging the body's immune system to specifically target and destroy cancerous cells. Specific monoclonal antibodies like trastuzumab, bevacizumab, pertuzumab, ertumaxomab, and atezolizumab are undergoing rigorous investigation for their therapeutic applications in breast cancer, including the challenging subset of triple-negative breast cancer [39]. By targeting specific molecular pathways and receptors, these monoclonal antibodies offer potential avenues for improving outcomes in breast cancer patients, particularly those with aggressive and treatment-resistant forms of the disease. Figure 1 shows the therapeutic applications of monoclonal antibodies.

Clinical successes and challenges

*FDA-Approved Monoclonal Antibodie*s

The FDA has played a pivotal role in the approval of numerous monoclonal antibodies for therapeutic use, marking significant milestones in the realm of medicine. One of the earliest FDA-approved monoclonal antibodies was muromonab-CD3 (Ortho clone OKT3) in 1986, formulated with a low protein concentration of 1 mg/mL [41]. Since then, the FDA has continued to expand its approval repertoire, with at least 103 antibody drugs sanctioned for marketing as of October 2021 [41]. These antibodies span a diverse array of indications and formats, encompassing innovative approaches like antibody-drug conjugates. Moreover, in response to the COVID-19 pandemic, the FDA has authorized the emergency use of monoclonal antibodies for the treatment of the virus. Bamlanivimab and etesevimab received emergency use authorization for the treatment of mild to moderate COVID-19 in non-hospitalized adults at high risk for disease progression [42]. These antibodies have demonstrated promising efficacy in reducing COVID-19-related hospitalizations and fatalities when administered in tandem. The swift authorization and deployment of monoclonal antibodies against COVID-19 underscore their critical role in combating emergent health threats. Overall, the development and approval of monoclonal antibodies have exerted a profound impact on modern medicine, furnishing effective treatments for a myriad of diseases and conditions. Their versatility and therapeutic efficacy continue to drive advancements in medical science, offering hope for improved patient outcomes and enhanced public health.

Safety and Efficacy Profiles

Efficacy: Monoclonal antibodies have garnered significant clinical acclaim, with over 20 mAbs currently in clinical use and a projected market value expected to reach $300 billion by 2025 [43,44]. Notably, specific mAbs have demonstrated notable benefits in the context of COVID-19, including reduced mortality risk, decreased reliance on mechanical ventilation, and facilitated hospital discharge [43,44]. However, in Alzheimer's disease, the efficacy of anti-amyloid-β mAbs remains ambiguous, with clinical trials yielding mixed results [45]. While some studies have shown promise, others have failed to demonstrate significant clinical benefits.

Safety: Despite their efficacy, monoclonal antibodies can be associated with adverse events, including immunogenicity, hypersensitivity reactions, and other toxicities [46]. However, the overall safety profile of mAbs appears favorable, with a large study of COVID-19 mAb treatments reporting that only 0.2% of patients experienced any adverse events [43]. Of particular note is the safety and efficacy of mAbs in immunocompromised patients, who may be more susceptible to drug interactions or contraindications with other therapies [43]. Thus, while mAbs offer significant therapeutic potential, careful consideration of safety profiles and patient characteristics is paramount in optimizing their clinical utility.

Challenges in Manufacturing and Cost

The manufacturing of monoclonal antibodies (mAbs) poses several challenges that affect both production capacity and cost. A significant obstacle lies in the limitations of current manufacturing and purification processes, which hinder the efficient production of therapeutic antibodies, thereby leading to increased costs [47]. These challenges have spurred the exploration of novel approaches, such as genetic delivery of therapeutic mAb genes for in vivo production, as a potential solution to enhance therapeutic efficacy and mitigate manufacturing costs [47]. Moreover, the intricacies inherent in the mAb manufacturing process contribute to production costs being overlooked. Factors such as the selection of raw materials and the optimization of manufacturing processes wield considerable influence over the overall cost of mAb production, underscoring the imperative for efficient and cost-effective manufacturing strategies [48]. In this regard, continuous flowsheets in mAb manufacture have emerged as a potential solution to overcome challenges and yield cost savings. Continuous manufacturing processes promise to reduce the cost of goods by 20% to 40% compared to traditional batch processes, thereby presenting an opportunity to optimize production efficiency and enhance cost-effectiveness in mAb manufacturing [49].

Future perspectives

Next-Generation Monoclonal Antibodies

Monoclonal antibodies hold immense promise for bolstering defenses against biological warfare agents and emerging infectious diseases [50]. Their hallmark traits include high specificity, the capacity to recruit the host immune system, the provision of rapid immunity, and a low incidence of adverse reactions [50]. Scientific evidence underscores the potential efficacy of mAbs in treating diseases instigated by biological warfare agents and natural pathogens that are of concern to the military [50]. Noteworthy advancements in antibody engineering have yielded more human-like mAbs with diminished immunogenicity, including chimeric, humanized, and fully human antibodies [50-52]. These next-generation mAbs are being cultivated for a wide spectrum of indications, ranging from cancer to autoimmune and infectious diseases [50-52]. The projected growth of the global therapeutic monoclonal antibody market to $300 billion by 2025 reflects the burgeoning expansion and future promise of this groundbreaking therapeutic paradigm [4]. Continued innovation in antibody engineering, coupled with the integration of genomic and medical data, alongside comprehensive clinical trials examining mAbs both in isolation and in conjunction with other therapies, will be pivotal in further augmenting their therapeutic efficacy [50-52]. Such concerted efforts hold the key to unlocking the full potential of monoclonal antibodies in revolutionizing disease treatment and prevention.

Personalized Medicine and Targeted Therapies

Personalized medicine and targeted therapies stand as pioneering approaches in contemporary healthcare, particularly within the sphere of oncology. Personalized medicine entails tailoring medical interventions based on an individual's genetic makeup and unique characteristics, thereby enabling more precise and efficacious treatments with diminished side effects [53,54]. Also known as precision medicine, this approach leverages genetic information to craft treatments that specifically target the distinct genetic attributes of a patient's cancer, resulting in enhanced outcomes and mitigated adverse effects [53]. Conversely, targeted therapies zero in on specific genes and proteins pivotal in the growth and survival of particular cancers. By pinpointing these targets, researchers can devise drugs that selectively act on these molecular pathways, furnishing more potent and customized treatments for various cancer types [51-53]. Targeted therapies have spearheaded advancements in cancer treatment, such as in breast cancer, where therapies directed at HER2 have yielded notable success and laid the groundwork for analogous approaches in other conditions like chronic myelogenous leukemia [53]. Both personalized medicine and targeted therapies represent the vanguard of modern oncology, marking a paradigm shift from conventional one-size-fits-all treatments to individualized and precise interventions. These transformative approaches hold immense promise for enhancing patient outcomes and quality of life, epitomizing the ethos of patient-centered care in the realm of cancer treatment [51-53].

Integration With Immunotherapy and Gene Therapy

The integration of monoclonal antibodies (mAbs) with immunotherapy and gene therapy signifies a cutting-edge approach in modern medicine, presenting innovative strategies to augment therapeutic outcomes. Immunotherapies centered around immune checkpoints (ICs) function by either blocking or stimulating pathways to bolster the immune system's capacity to identify and combat diseases, particularly cancer [19]. When combined with mAbs, these immunotherapeutic regimens exhibit heightened efficacy, resulting in improved patient responses and outcomes. Furthermore, gene therapy has garnered attention as an alternative avenue for producing mAb therapeutics, offering a promising solution to circumvent the challenges associated with traditional mAb production and administration [54]. Gene therapy entails the delivery of genes encoding mAbs of interest, thereby facilitating in vivo mAb production within the body's cells. This innovative approach obviates the need for conventional mAb production and purification processes, potentially reducing costs and enhancing accessibility to these life-saving treatments [54]. The convergence of mAbs, immunotherapy, and gene therapy heralds new frontiers in personalized and targeted treatments across a spectrum of diseases, encompassing cancer and autoimmune disorders. By harnessing the complementary strengths of each approach, researchers and clinicians are advancing towards more efficacious and streamlined therapeutic interventions that hold immense promise for the future of medicine.

## Conclusions

In conclusion, the evolution of monoclonal antibodies (mAbs) has been a transformative journey within the realm of modern medicine. Beginning with their discovery and subsequent development of technologies such as hybridoma and recombinant DNA, mAbs have emerged as powerful therapeutic agents with diverse applications across various medical fields. Their precise targeting capabilities and mechanisms of action have revolutionized the treatment landscape, offering effective solutions for conditions ranging from cancer to autoimmune disorders and infectious diseases. Looking forward, the future of mAbs holds immense promise, with ongoing research focusing on next-generation therapies, personalized medicine approaches, and integration with emerging modalities like immunotherapy and gene therapy. However, challenges related to manufacturing scalability, affordability, and equitable access to treatments persist, underscoring the need for continued innovation and collaboration. Nevertheless, the remarkable journey of monoclonal antibodies stands as a testament to human innovation and dedication to advancing healthcare, paving the way for a future where targeted and effective therapies offer hope and healing to patients worldwide.
